# Deletion of lymphotoxin-β receptor (LTβR) protects against acute kidney injury by PPARα pathway

**DOI:** 10.1186/s10020-024-01026-z

**Published:** 2024-12-20

**Authors:** Zufeng Wang, Yichun Cheng, Jiahe Fan, Ran Luo, Gang Xu, Shuwang Ge

**Affiliations:** 1 Department of Nephrology, Tongji Hospital, Tongji Medical College, Huazhong University of Science and Technology, No.1095 Jiefang Road, Wuhan, 430030 China; 2 Department of Nephrology, The First Affiliated Hospital of Guangxi Medical University, Nanning, 530021 China

**Keywords:** Acute kidney injury, Lymphotoxin-β receptor, PPARα, NF-κB, Apoptosis

## Abstract

**Background:**

Recent data has shown a considerable advancement in understanding the role of lymphotoxin-β receptor (LTβR) in inflammation. However, the functions and underlying mechanisms of LTβR in acute kidney injury (AKI) remain largely unknown.

**Methods:**

AKI was induced in mice by renal ischemia-reperfusion (I/R). HK-2 cells and primary renal tubular epithelial cells (RTECs) were subjected to hypoxia/reoxygenation (H/R) injury. The effects of LTβR depletion were examined in mice, as well as primary RTECs. Bone marrow chimeric mice was generated to determine whether the involvement of LTβR expression by parenchymal cells or bone marrow derived cells contributes to renal injury during AKI. RNA sequencing techniques were employed to investigate the mechanism via which LTβR signaling provides protection against I/R-induced AKI

**Results:**

LTβR expression was downregulated both in vivo and in vitro models of AKI. Moreover, depletion of LTβR decreased renal damage and inflammation in I/R-induced AKI. We also found that LTβR deficient mice engrafted with wild type bone marrow had significantly less tubular damage, implying that LTβR in renal parenchymal cells may play dominant role in I/R-induced AKI. RNA sequencing indicated that the protective effect of LTβR deletion was associated with activation of PPARα signaling. Furthermore, upregulation of PPARα was observed upon depletion of LTβR. PPARα inhibitor, GW6471, aggravated the tubular damage and inflammation in LTβR^−/−^ mice following I/R injury. Then we further demonstrated that LTβR depletion down-regulated non-canonical NF-κB and Bax/Bcl-2 apoptosis pathway through PPARα.

**Conclusions:**

Our results suggested that the LTβR/PPARα axis may be a potential therapeutic target for the treatment of AKI.

**Supplementary Information:**

The online version contains supplementary material available at 10.1186/s10020-024-01026-z.

## Background

Acute kidney injury (AKI) is a common medical complication, and its incidence has progressively increased worldwide, imposing significant medical and economic burdens on the healthcare system (Levey and James 2017; Hoste et al. 2018). AKI is associated with high morbidity and mortality, particularly in intensive care units, where in-hospital mortality rates for AKI have been reported to exceed 50% (Hoste et al. 2015). Although a patient may survive the acute illness, AKI increases the risk of developing chronic kidney disease and accelerates progression to end-stage renal disease (Chawla et al. 2014). Despite decades of research, there is currently no effective therapeutic option for AKI.

The lymphotoxin-β receptor (LTβR), a member of the tumor necrosis factor receptor superfamily, is originally identified as a key mediator in the development and homeostasis of lymphoid tissues and organs (Fütterer et al. 1998). In recent years, an increasing body of literature has reported that LTβR signaling is broadly activated during inflammation (Gubernatorova and Tumanov 2016). Blocking LTβR signaling through gene deficiency or the use of neutralizing antibodies has been shown to be beneficial in various inflammatory and autoimmune diseases, including colitis, arthritis, autoimmune pancreatitis, and experimental autoimmune encephalomyelitis (Columba-Cabezas et al. 2006). A recent study reported that LTβR protein is localized to renal tubular epithelial cells (RTECs), and LTβR blockade significantly improved renal function in a murine lupus model (Seleznik et al. 2016). Ischemia/reperfusion (I/R) is a leading cause of AKI, particularly in patients undergoing major surgical procedures (Han and Lee 2019). Due to their high metabolic rate, RTECs are highly sensitive to damage following renal I/R (Chevalier 2016). RTECs necrosis is a hallmark of AKI, resulting in the subsequent release of inflammatory and vasoactive mediators (Jang and Rabb 2015). Therefore, LTβR signaling may play an essential role in I/R induced AKI.

The downstream regulatory mechanisms of LTβR remain to be fully elucidated. Recent reports have indicated that non-canonical NF-κB signaling is involved in LTβR-mediated inflammation (Madge et al. 2008). This pathway encompasses the NF-κB inducing kinase (NIK)-dependent processing of p100 and the formation of the RelB/p52 NF-κB complex, which leads to nuclear translocation and transcription of target genes (Mitchell and Carmody 2018). A previous study demonstrated that NIK was activated in blood vessels and regulated inflammation-induced angiogenesis in response to LTβR (Noort et al. 2014). In rheumatoid arthritis, sustained activation of LTβR triggers non-canonical NF-κB signaling, promoting the expression of inflammatory mediators and adhesion molecules in vascular endothelial cells (Kucharzewska et al. 2019). However, the role of LTβR and its underlying mechanism in AKI are less well defined. The current study aimed to investigate whether the deletion of LTβR alters recovery from AKI and to explore the underlying mechanism.

## Methods

### Mice

C57BL/6J mice (age, 4w and 8w; weight, 20-25 g) were purchased from Beijing Vital River Laboratory Animal Technology Co., Ltd (Beijing, China). LTβR^−/−^ mice (age, 4w and 8w; weight, 20-25g) were purchased from Baiaosaitu Biotechnology Co., LTD (Beijing, China). All mice were maintained in specific pathogen-free conditions at Tongji Medical College of Huazhong University of Science and Technology. All animal experiments were approved by the Experimental Animal Ethics Committee of Tongji Hospital, Tongji Medical College, Huazhong University of Science and Technology (TJH-202111023).

### Patients

In order to identify the expression of LTβR in AKI patients. We chose 5 AKI patients and 5 minimal change disease (MCD) patients. The patients who meet one of the following criteria were identified as AKI:(a) serum creatinine (Scr) increased more than 26.5 μmol/L within 48 h; (b) 50% increase in Scr within 1 week; (c) urine output less than 0.5 mL/(kg·h) and last more than 6 h. The MCD patients diagnosed by renal pathology with minimal lesions. The baseline data of these patients was showed in Table S1. This study was approved by the ethics Committee of Tongji Hospital, Tongji Medical College, Huazhong University of Science and Technology (TJ-IRB20220501), and informed consent was obtained from the patients.

### Immunohistochemistry

Paraffin-embedded kidneys of mice were cut into 4 μm sections. Citrate buffer (PH 6.0) was used for antigen retrieval for 25 min after deparaffinization and rehydration. Then, the kidney sections were blocked by 10% H_2_O_2_ for endogenous peroxidase for 15 min and blocked with 5% serum for secondary antibodies for 30 min at room temperature. Primary antibody against LTβR (1:150, rabbit, DF7292; Affinity, China) was used to incubate the kidney tissue overnight at 4 °C. Horseradish peroxidase (HRP)-conjugated antibodies were used to treat the sections the next day. 3,3′-diaminobenzidine (DAB) (G1215-200T, Servicebio, China), as an HRP-specific substrate, was used to show the specific stained area of kidney tissue. Then, Image J (NIH, USA) was used to measure the average optical density of images to show the expression of LTβR.

### Histology

Paraffin-embedded kidneys of mice were cut into 4 μm sections. Renal pathology was evaluated by performing Periodic Acid-Schiff (PAS) staining and hematoxylin and eosin (H&E) staining. The damage of kidney was evaluated by ten randomly selected fields. The score system was as follows: 1: 1–10% kidney tubular damage; 2: 11–25% kidney tubular damage; 3: 26–50% kidney tubular damage; 4: 51–75% kidney tubular damage; 5: 76–100% kidney tubular damage.

### Real-time PCR

Trizol reagent was used to extract the total RNA from the kidneys and cells according to the manufacturer’s instructions (Invitrogen, USA). Template cDNA was obtained using a reverse transcription system kit (Vazyme, China). Real time-quantitative polymerase chain reaction (RT-qPCR) was measured as polymerase chain carried out using the SYBR master-mix (Vazyme, China). The expression levels were normalized by GAPDH level. The designed primers are listed in supplemental Table S2.

### Animal model

The procedure of ischemic reperfusion (I/R) injury model was as follow: mice were anesthetized with a dose of 35-40 mg/kg of 1% sodium pentobarbital solution (10 μL/g, Sigma, USA) by intraperitoneal injection. Both renal pedicles were exposed through flank incisions and were clamped with an atraumatic vascular clip for 28 minutes (Roboz Surgical Instrument Co., Germany). Upon removal of the clamp, return of blood flow was confirmed visually, and then the incision was sutured. Analgesia was achieved through the subcutaneous injection of 0.5% bupivacaine at the wound site, administered both during and after surgery with a dosage of 1mg/kg. The mice were euthanized 1, 3, 5, 7 days after the operations. Sham group were subjected to a similar surgical procedure without clamping renal pedicles.

### Serum biochemical analyses

Blood urea nitrogen (BUN) and Scr of mice was measured according to the manufacturer’s protocols (Nanjing Jiancheng Biology Engineering Institute, Nanjing, China).

### Transcriptome sequencing and bioinformatics analysis

Total RNA was isolated from kidney tissue and subjected to cDNA synthesis, fragmentation, adapter ligation, and PCR amplification. Illumina HiSeq platform was used to perform the Sequencing. The differentially expressed genes (DEGs) were obtained by the platform of BGI (fold change ≥ 1 and adjusted P value ≤ 0.05). Kyoto Encyclopedia of Genes and Genomes (KEGG) analysis was performed by the platform of BGI as well.

### Cell culture

HK-2 cells were cultured in DMED/F12 with 10%FBS. The primary RTECs were extracted from mice kidneys. Kidneys were removed from normal mice under sterile conditions, minced, and digested with collagenase-IV for 1 h at 37 °C. The digestion was terminated with a complete medium and the suspension was filtered through two cell strainers of 40 μm and 70 μm. Then the suspension was centrifuged at 1200 rpm for 10 min. After cracking of red blood cells and washing twice, RTECs were cultured in a cell culture dish in DMEM/F12 supplemented with 10% FBS (Gibco, USA), 1% penicillin, and streptomycin and other growing factors.

The cells were deprived of oxygen for 24 h, followed by reoxygenation for 1 h, 3 h, 6 h, and 12 h. The hypoxia/reoxygenation(H/R) procedures are as follows: the cells were rinsed with sterile PBS, and cultured with the sugar-free medium, and placed in the hypoxia incubator (1%O_2_); After 24 h of hypoxia treatment, the cells were cultured with the normal complete medium in a normoxia 37 °C incubator for different duration.

### Western Blot

RIPA lysis buffer with protease inhibitor was used to lysis renal tissues and cells. The 10% SDS-PAGE was used to load the protein, and equal amounts of protein(50ug) from each sample were loaded. Then the protein was transferred to polyvinylidene fluoride membranes (Millipore, USA). 5% skimmed milk dissolved in Tris Buffered saline Tween (TBST) solution was used to block the membranes for 1h. After that, primary antibodies against LTβR (1:1000, rabbit, DF7292; Affinity, China), P100/P52(1:1000, rabbit, 4882S, Cell Signaling Technology, USA), NIK(1:1000, rabbit, 4994S, Cell Signaling Technology, USA), RelB(1:1000, rabbit, 10544S, Cell Signaling Technology, USA), PPARα(1/200, mouse, sc-398394; Santa Cruz Biotechnology, Texas), and β-Actin(1:10000, AC026, rabbit; Abclonal, China) were used to incubate the membranes at 4 ℃ overnight. After that, TBST was used to wash the membranes 3 times. Then the membranes were incubated with anti-rabbit-IgG-HRP-conjugate (1:2000, 7074S, goat; Cell signaling Technology, USA) or anti-mouse-IgG-HRP-conjugate (1:2000, 7056S, goat; Cell signaling Technology, USA) diluted in 5% nonfat milk in TBST for 1h. The blots were visualized by hypersensitive exposure solution (G2020-25ML, Servicebio, China). The relative expression of target molecules was normalized to the internal reference protein and quantified by using ImageJ (NIH, USA).

### TUNEL assay

In order to detect the apoptotic cells in cultured cells and the paraffin section, a TUNEL assay was used. Briefly, the detection of apoptotic cells was displayed according to the instructions of the TUNEL kit (GDP1042, Servicebio, China). After the detection, the images were acquired using a Nikon TE 2000 fluorescence microscope (Nikon, Japan). Then, we randomly selected ten high-powered fields from each section to calculate the number of apoptotic cells.

### Compound administration

GW6471 (HY-15372, Med Chem Express, USA) was dissolved in DMSO according to the illustration of this compound. The drug was given by intraperitoneal injection with the dosage of 10 µg/g. The cultured cells were pretreated with GW6471 for 1 h (25 μM) before the H/R treatment.

### Bone marrow transplantation

The recipient mice were feed with antibiotic-supplemented water (5 mM sulfamethoxazole, 0.86 mM trimethoprim) for 1 week prior to irradiation and maintained for 2 weeks after irradiation. Then the recipient mice were anesthetized by peritoneal injection of 1% pentobarbital (10 μL/g, Sigma, USA) and received a total radiation dose of 11 Gy.

The donor mice were euthanized and put in the 75% alcohol for 10 mins. Then the joints and the ends of the long bones was obtained. The marrow from the long bone was obtained. After the lysis of red cells and centrifugation. 5 × 10^6^ bone marrow cell were transplanted to the recipient mice within 4 h after irradiation. Six weeks later, the bone marrow reconstituted recipients underwent I/R operation. All animal experiments in this section were approved by the Experimental Animal Ethics Committee of Huazhong University of Science and Technology (S2723).

### Flow cytometry

Kidneys were isolated from mice and the cell suspension of kidneys was obtained using collagenase IV. Afterwards, the cell suspension was divided into several tubes and incubated primary antibody for 30 minutes. Following antibodies were used from BioLegend for flow cytometry analysis: BV510-conjugated anti–mouse Zombie dye, APC/Cy7-conjuated anti-mouse CD45 antibody, PE conjugated anti-mouse CD11b antibody, PE/Cy7-conjugated anti–mouse Ly6G antibody; APC -conjugated anti–mouse F4/80 antibody. The gating strategy was showed in Figure S1.

### Statistical analysis

Numbers of animals of each group were showed in each figure legend. We used the one-way ANOVA test, unpaired t test or Mann-Whitney test to compare the difference between groups. A value of P < 0.05 was assumed to be statistical significant. Statistical analysis was carried out using SPSS 23.0 software (SPSS, USA) and GraphPad Prism version 6 software (Graph software, San Diego, CA).

## Results

### LTβR expression is decreased in kidney following AKI

**Fig. 1:**
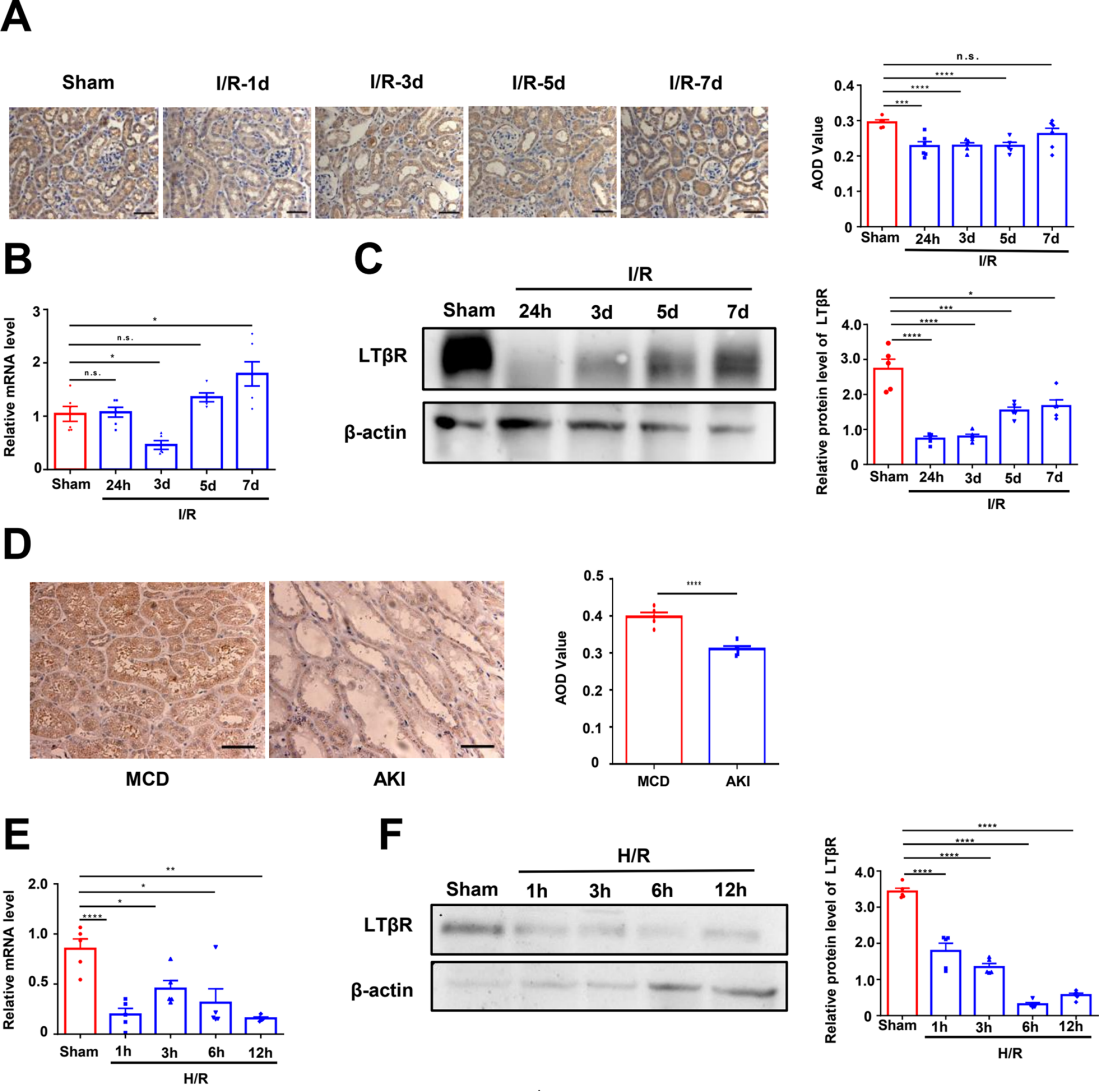
LTβR is downregulated in the kidney after I/R injury. In animal experiments, kidneys were harvested at various time points during reperfusion after ischemia. **A**–**C** The representative images of immunohistochemical analysis of LTβR (**A**), relative mRNA levels (**B**), and relative protein expression (**C**) of LTβR in kidneys from different groups of WT mice showed the expression of LTβR was reduced after I/R compared with sham groups and got gradually recovered during 7d. (Sham, I/R-24h, I/R-3d, I/R-5d, and I/R-7d, n = 5/group). **D** Representative images of immunohistochemical stained of LTβR in kidneys showed a decreased expression of LTβR in MCD patients compared with AKI patients (n = 5/group). **E**, **F** Reduced relative mRNA levels (**E**) and protein expression (**F**) of LTβR in HK-2 cells from H/R groups compared with sham groups (Sham, H/R-1h, H/R-3h, H/R-6h, and H/R-12h, n = 5/group). Scale bar, 50 μm. Graph data were presented as mean ± SEM. Each point represents an individual subject. P values were determined by t-test. *p < 0.05, **p < 0.01, ***p < 0.005, ****p < 0.001

Initially, we evaluated the expression of LTβR in the kidney following I/R damage. We found that renal tubules had extensive expression of LTβR. Moreover, the expression of LTβR was significant reduced 24h after I/R injury, and then gradually recovered but still lower than control at 7th day (Fig. 1A). Similar patterns in the mRNA and protein level were also observed (Fig. 1B and C). We further evaluated LTβR expression in HK-2 cells after H/R injury and in kidney biopsies from AKI patients. We found that there was a decrease in LTβR expression in kidney biopsy samples from AKI patients compared to MCD patients (Fig. 1D). Consistently, the expression of LTβR was also reduced in HK-2 cells after H/R injury (Fig. 1E and F).

### Deletion of LTβR attenuate I/R induced AKI in mice

**Fig. 2:**
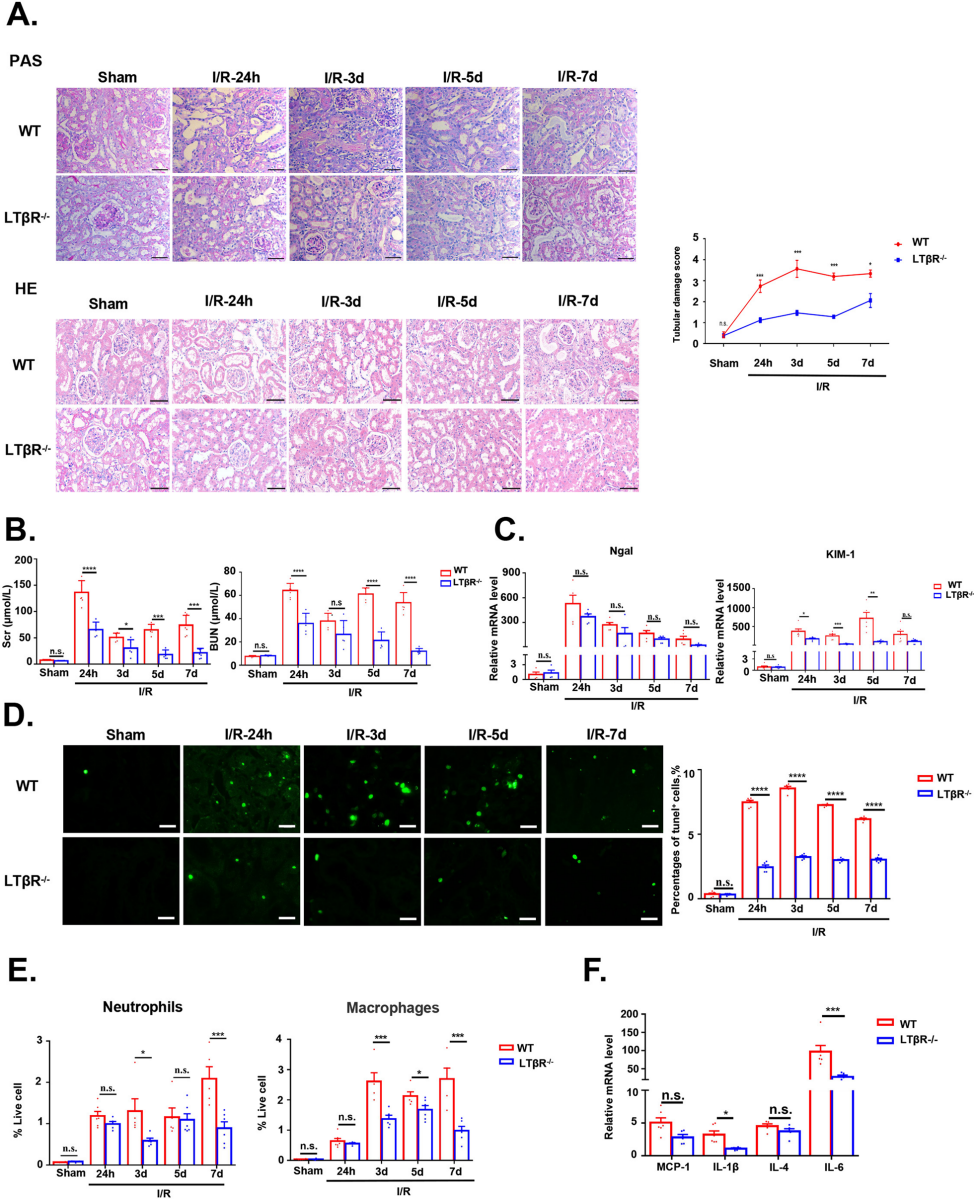
Depletion of LTβR alleviates kidney damage in I/R injury. **A** Representative images of PAS-stained and HE-stained of kidneys from WT mice and LTβR^−/−^ mice in different groups. Reduced tubular damage score of kidneys from LTβR^−/−^ mice compared with WT mice in I/R groups. **B** Scr and BUN of WT mice and LTβR^−/−^ mice in different groups. **C** Relative RNA levels of Ngal and KIM-1 in kidneys from LTβR^−/−^ mice and WT mice in different groups. **D** Representative images of TUNEL stained of kidneys from WT mice and LTβR^−/−^ mice in different groups. Fewer cell deaths in LTβR^−/−^ mice compared with WT mice in I/R groups. **E**, **F** LTβR deficiency relieved inflammation of kidneys after I/R injury. **E** Neutrophils and macrophages infiltration in kidneys from LTβR^−/−^ mice and WT mice. **F** Relative RNA level of inflammation factors in kidneys from LTβR^−/−^ mice and WT mice in the I/R-24h group. Scale bar, 50 μm. WT: Sham, n = 6; I/R-24 h, n = 6; I/R-3d, n = 5; I/R-5d, n = 5; I/R-7d, n = 5. LTβR^−/−^: Sham, n = 5; I/R-24 h, n = 5; I/R-3d, n = 5; I/R-5d, n = 6; I/R-7d, n = 6. Graph data were presented as mean ± SEM. Each point represents an individual subject. P values were determined by t-test. *p < 0.05, **p < 0.01, ***p < 0.005, ****p < 0.001

To evaluate the effect of LTβR in AKI, we subjected LTβR^−/−^ mice and corresponding WT mice, to I/R or sham operation. Our findings demonstrated that, in comparison to WT mice, the tubular damage score was much lower in LTβR^−/−^ mice and the loss of brush boundary in the renal tubular epithelial cells caused by I/R was significantly mitigated (Fig. 2A). I/R-induced increases in Scr and BUN were also attenuated in LTβR^−/−^ mice (Fig. 2B). Moreover, mRNA level of Ngal and KIM-1, markers of kidney injury, were also decreased in LTβR^−/−^ mice (Fig. 2C). TUNEL staining showed that there were less apoptosis cells in LTβR^−/−^ mice as well (Fig. 2D).

Since inflammatory response plays a key role in the pathogenesis of AKI, then we compared the inflammatory cell and mediators between LTβR^−/−^ and WT mice. Ly6G-positive neutrophil and F4/80-positive macrophages were measured by flow cytometry. Our results showed that there was less infiltration of neutrophil and macrophages in LTβR^−/−^ mice compared with WT mice (Fig. 2E). Moreover, increasing of inflammatory cytokines, such as IL-1β, IL-4, IL-6 and MCP-1, were suppressed in LTβR^−/−^ mice as well (Fig. 2F). Therefore, our data provided evidences that deficiency in LTβR protected against kidney injury and inflammatory reactions response by I/R.

### LTβR signaling in renal tubular epithelial cells contributed to I/R injury in mice

**Fig. 3:**
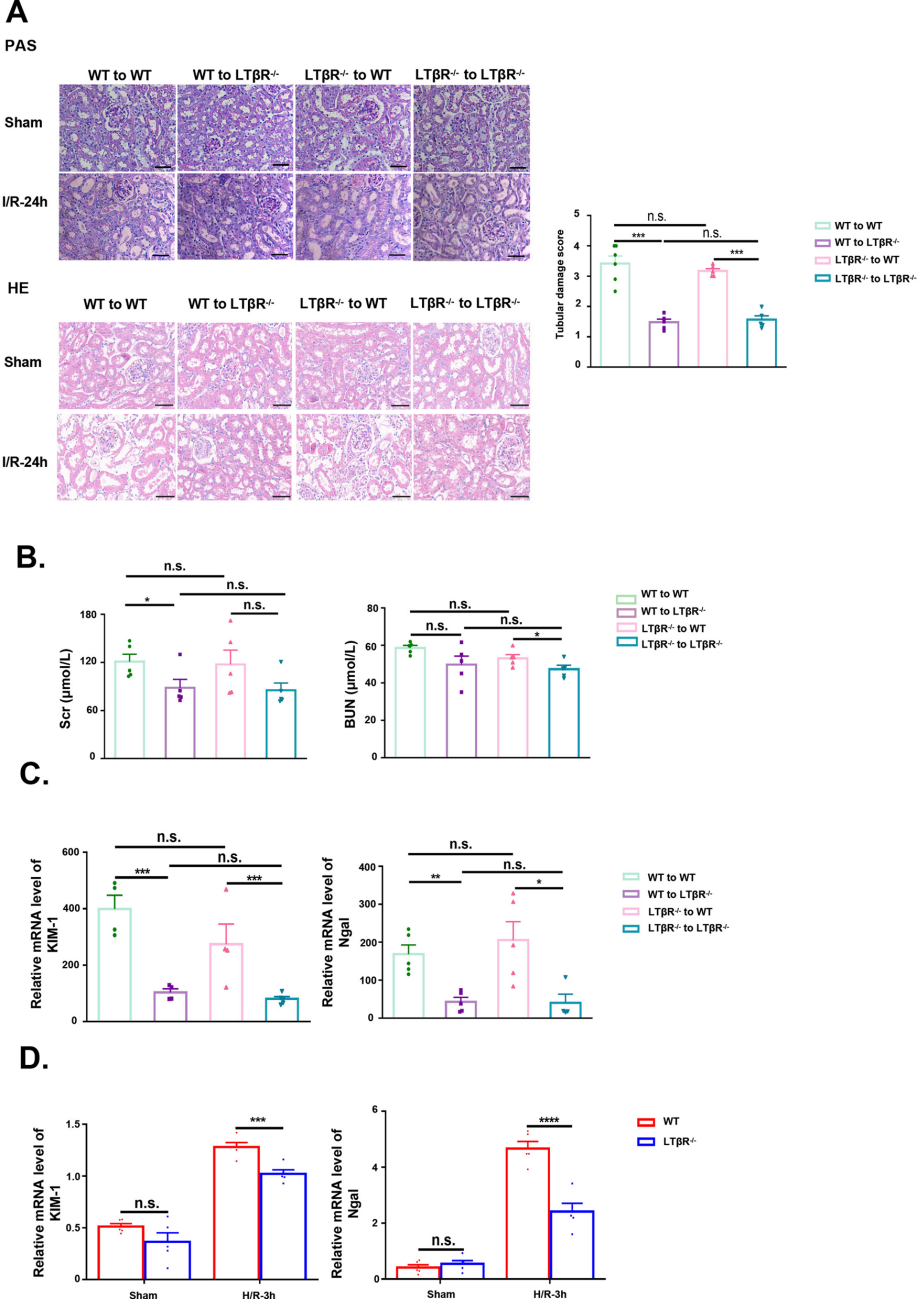
The injury of kidneys in different bone marrow transplantation groups after I/R-24h. **A**–**C** showed the loss of LTβR in renal parenchymal cell protect kidneys after I/R. **A** Representative images of PAS-stained and HE-stained of kidneys from different bone marrow transplantation (BMT) groups after I/R. The graph showed the tubular damage score of kidneys from different BMT groups after I/R. **B** Scr and BUN of mice from different BMT groups after I/R. **C** Relative RNA level of kidney damage molecules (Ngal and KIM-1) of kidneys from different BMT groups after I/R. N = 5/group. **D** Relative RNA level of Ngal and KIM-1of RTEC from WT and LTβR^−/−^ mice in sham and H/R group. N = 5/group. Scale bar, 50 μm. Graph data were presented as mean ± SEM. Each point represents an individual subject. P values were determined by t-test. *p < 0.05, **p < 0.01, ***p < 0.005, ****p < 0.001

Previous studies reported that LTβR was both expressed on dendritic cells, macrophages, and epithelial cell, we next determined the relative importance of LTβR signaling via bone marrow–derived cells or renal parenchymal cells in the pathogenesis of renal I/R by BMT. Scr levels and tubular injury in bone marrow chimeras were similar to that observed in control mice, indicating there was no damage of the BMT procedure on the kidney (Fig. S2A–C). Notably, compared to WT to WT chimeras, WT to LTβR^−/−^ chimeras, which lacked LTβR in renal parenchymal cell, showed significantly ameliorated I/R induced AKI, assessed by Scr level and tubular damage, to a degree similar to LTβR^−/−^ to LTβR^−/−^ mice. Whereas LTβR^−/−^ to WT chimeras showed no difference compared with WT to WT chimeras (Fig. 3A–C). Given that LTβR was wildly expressed on renal tubules, we cultured primary RTEC treated with H/R injury to further identify whether LTβR deficiency protects renal tubular cells against AKI. Primary RTECs from mice kidney were verified using IF staining of CK18 (Fig. S2D). We found that the primary RTECs from LTβR^−/−^ mice demonstrated lower expression of NGAL and KIM-1 compared with primary RTECs from WT mice (Fig. 3D). These results suggested that LTβR in renal tubules play a key role in pathogenesis of AKI.

### LTβR deficiency activated PPARα in I/R induced AKI.

**Fig. 4:**
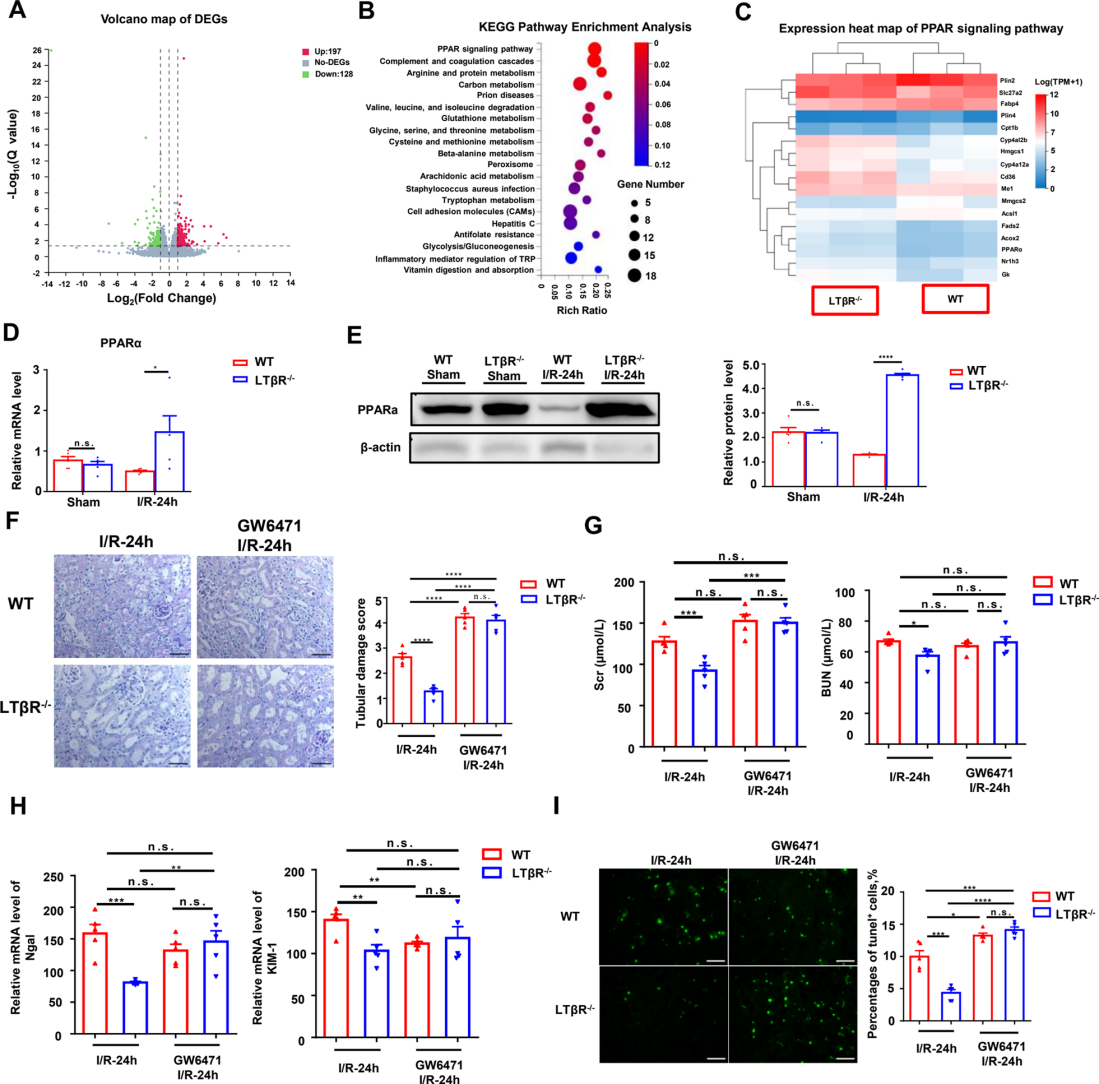
LTβR deficiency activated PPARα in I/R-induced AKI. **A**–**C** Transcriptome analysis of kidneys in WT and LTβR^−/−^ mice. **A** Volcano plot of gene expression changes between kidneys from WT and LTβR^−/−^ mice after I/R. 197 upregulated genes and 128 downregulated genes with fold change ≥ 1 and adjusted P value ≤ 0.05 were found in kidneys from LTβR^−/−^ mice compared with WT mice. **B** KEGG pathway functional enrichment analysis of all upregulated DEGs (FDR ≤ 0.01). **C** Heatmap of PPAR pathway (N = 3/group). **D**, **E** Relative RNA level (**D**) and protein level (**E**) of PPARα in kidneys from WT and LTβR^−/−^ mice. **F**–**I** Pretreated mice with a specific PPARα inhibitor, GW6471, eliminated the renoprotection of the loss of LTβR. **F** Representative images of PAS-stained of kidneys from WT mice and LTβR^−/−^ mice in control I/R group and pretreated group. Tubular damage score showed the kidney damage got worse after the pretreated of GW6471. **G** Scr and BUN of WT mice and LTβR^−/−^ mice in the control I/R group and pretreated group. H: Relative mRNA level of Ngal and KIM-1 of kidneys from WT mice and LTβR^−/−^ mice in the control I/R group and pretreated group. **I** Representative images of TUNEL stained of kidneys from WT mice and LTβR^−/−^ mice in different groups. More cell deaths in the pretreated group compared with the control I/R groups (N = 5/group). Scale bar, 50 μm. Graph data were presented as mean ± SEM. Each point represents an individual subject. P values were determined by t-test. *p < 0.05, **p < 0.01, ***p < 0.005, ****p < 0.001

In order to further clarify the mechanism underlying protective effect of LTβR in AKI, we used RNA sequencing to explore the DEGs and pathways between kidney of LTβR^−/−^ and WT mice following I/R injury. Our results showed there were 197 up-regulated genes and 128 down-regulated genes between LTβR^−/−^ mice and WT mice after I/R (Fig. 4A). KEGG pathway analysis demonstrated that DEGs are enriched in the PPAR pathway (Fig. 4B). We noted that the expression of PPARα, a member of PPAR subfamily, significantly up-regulated in LTβR^−/−^ mice compared with WT mice (Fig. 4C). Moreover, we confirmed the increased mRNA and protein level of PPARα in kidney from LTβR^−/−^ mice, while there was no difference between sham groups (Fig. 4D and E). Next, we used the specific PPARα inhibitor, GW6471, to confirm the role of PPARα in LTβR mediated protective effect in I/R injury. After treatment with GW6471, the tubular damage and renal function got worse in LTβR^−/−^ mice following I/R injury (Fig. 4F–G). Moreover, in primary RTECs pretreated with GW6471, the mRNA expression of KIM-1 and Ngal, as were as the cell apoptosis, were increased after H/R injury (Fig. 4H, I). Thus, these results suggested that LTβR deficiency regulated the activation of PPARα in protecting against AKI.

### LTβR regulate non-canonical NF-κB pathway via PPARα during I/R injury

**Fig. 5:**
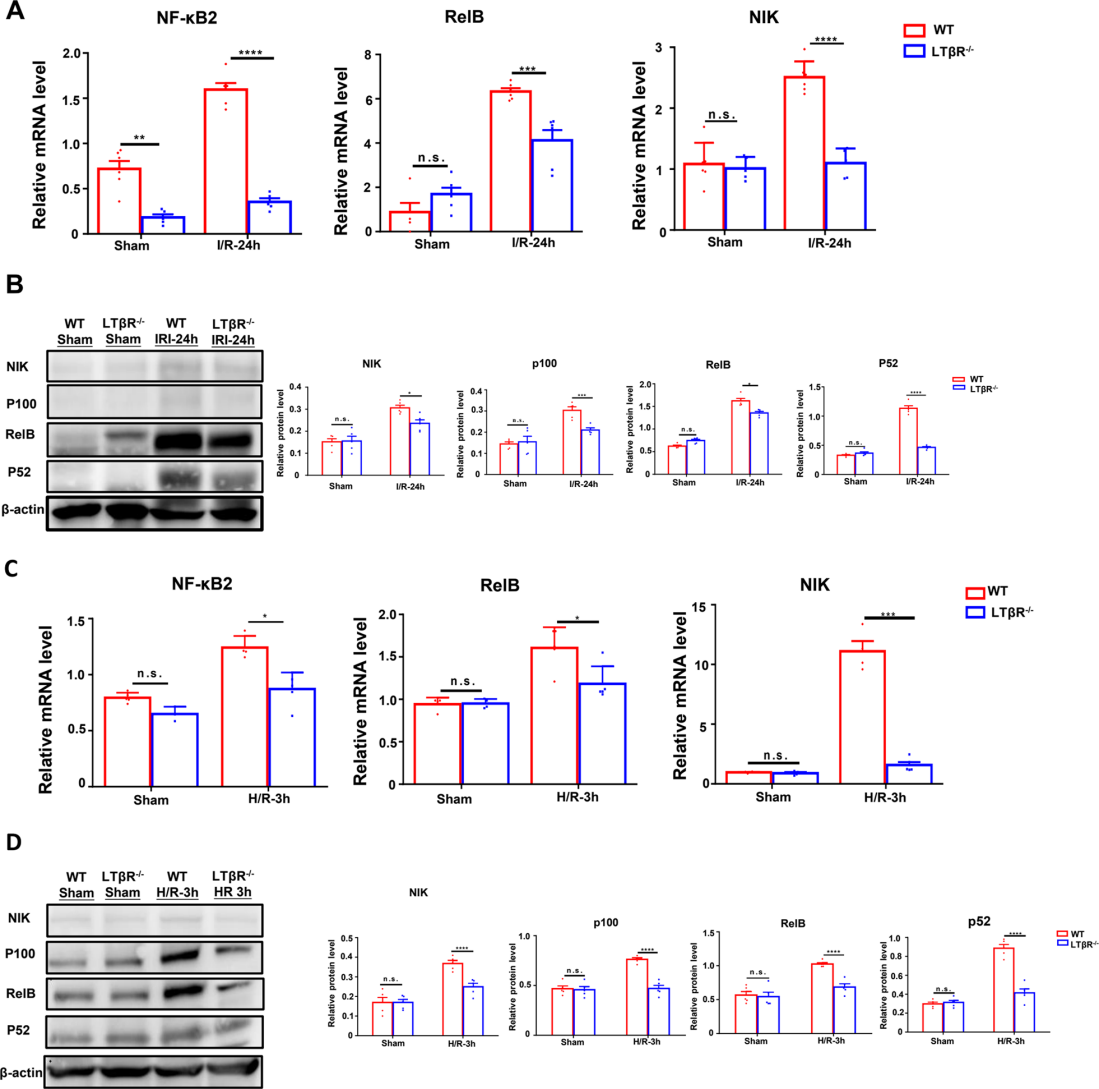
Loss of LTβR downregulated the non-canonical NF-κB pathway. Relative RNA level (**A**) and protein level (**B**) of NF-κB2, Relb, and NIK in kidneys from WT and LTβR^−/−^ mice in sham and I/R group. Relative RNA level (**C**) and protein level (**D**) of NF-κB2, Relb, and NIK in RTEC from WT and LTβR^−/−^ mice’ kidneys in sham and H/R group. N = 5/group. Graph data were presented as mean ± SEM. Each point represents an individual subject. P values were determined by t-test. *p < 0.05, **p < 0.01, ***p < 0.005, ****p < 0.001

It is reported that LTβR signaling mediates activation of non-canonical NF-κB pathway. Our results showed that the mRNA expression of NF-κB_2_, Relb, NIK, key molecules of non-canonical NF-κB pathway, was significantly decreased in LTβR^−/−^ mice compared with WT mice in I/R injury (Fig. 5A). Western blot analysis also confirmed the inhibition of non-canonical NF-κB pathway in LTβR^−/−^ mice (Fig. 5B). In addition, we observed consistent result in primary RTECs following H/R injury (Fig. 5C, D).

**Fig. 6:**
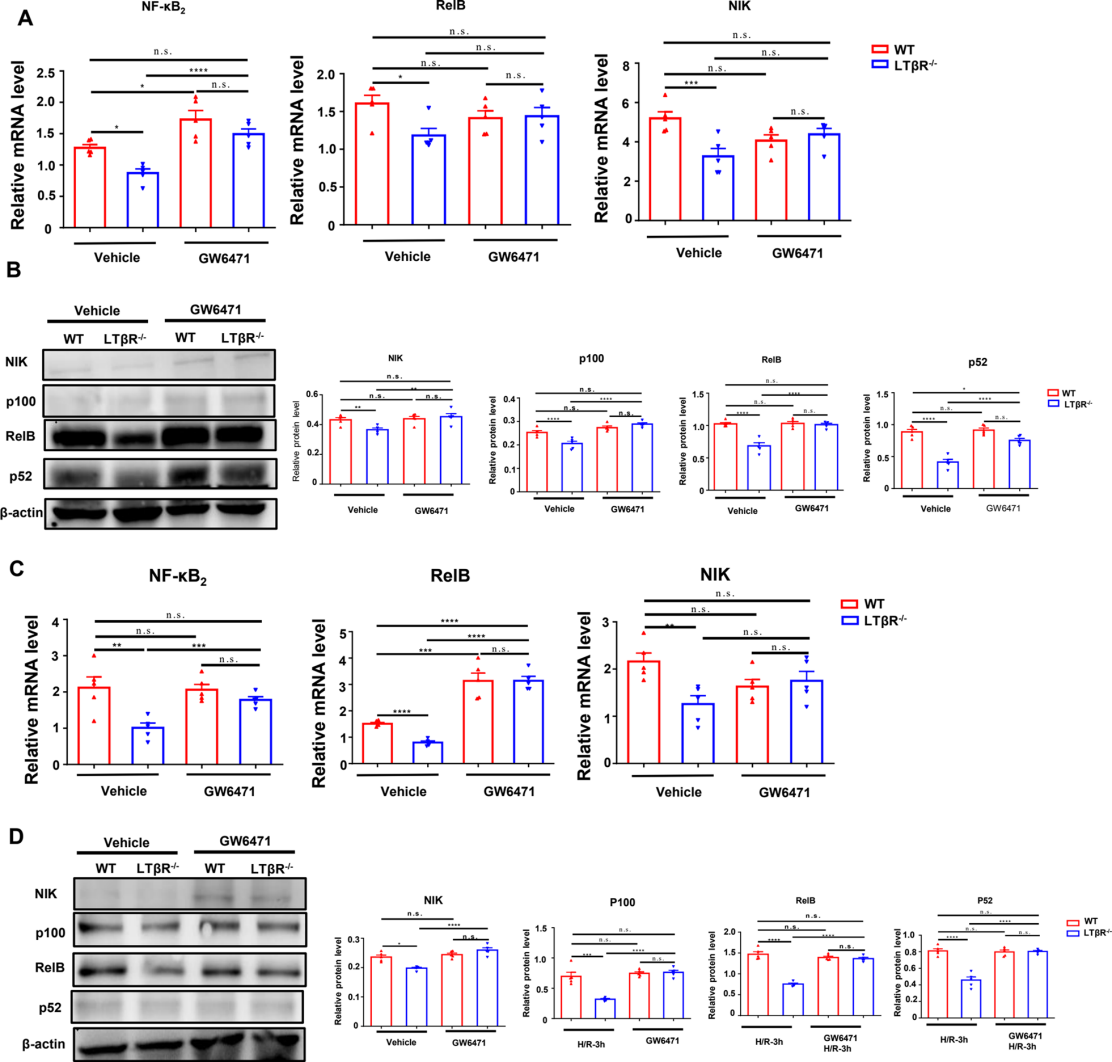
Specific PPARα inhibitor upregulated the non-canonical NF-κB pathway in kidneys from LTβR^−/−^ mice. Relative RNA level (**A**) and protein level (**B**) of NF-κB2, Relb, and NIK in kidneys from WT and LTβR^−/−^ mice in control I/R group and GW6471 pretreated group. Relative RNA level (**C**) and protein level (**D**) of NF-κB2, Relb, and NIK in RTEC from WT and LTβR^−/−^ mice’ kidneys in the control H/R group and GW6471 pretreated group. N = 5/group. Graph data were presented as mean ± SEM. Each point represents an individual subject. P values were determined by t-test. *p < 0.05, **p < 0.01, ***p < 0.005, ****p < 0.001

We have previously observed that the PPARα was a crucial regulator in LTβR mediated protective effect in I/R injury. We next sought to investigate whether PPARα participate in LTβR and non-canonical NF-κB pathway axis. Our results showed that after treatment with GW6471, the expression levels of key molecules of non-canonical NF-κB were increased in LTβR^−/−^ mice following I/R injury, compared with control group treated with vehicle (Fig. 6A–C). Similar results were observed in primary RTECs pretreated with GW6471 before H/R injury (Fig. 6C, D). These data suggest that LTβR can regulate non-canonical NF-κB pathway via PPARα.

### Deletion of LTβR attenuates I/R induced apoptosis via PPARα

**Fig. 7:**
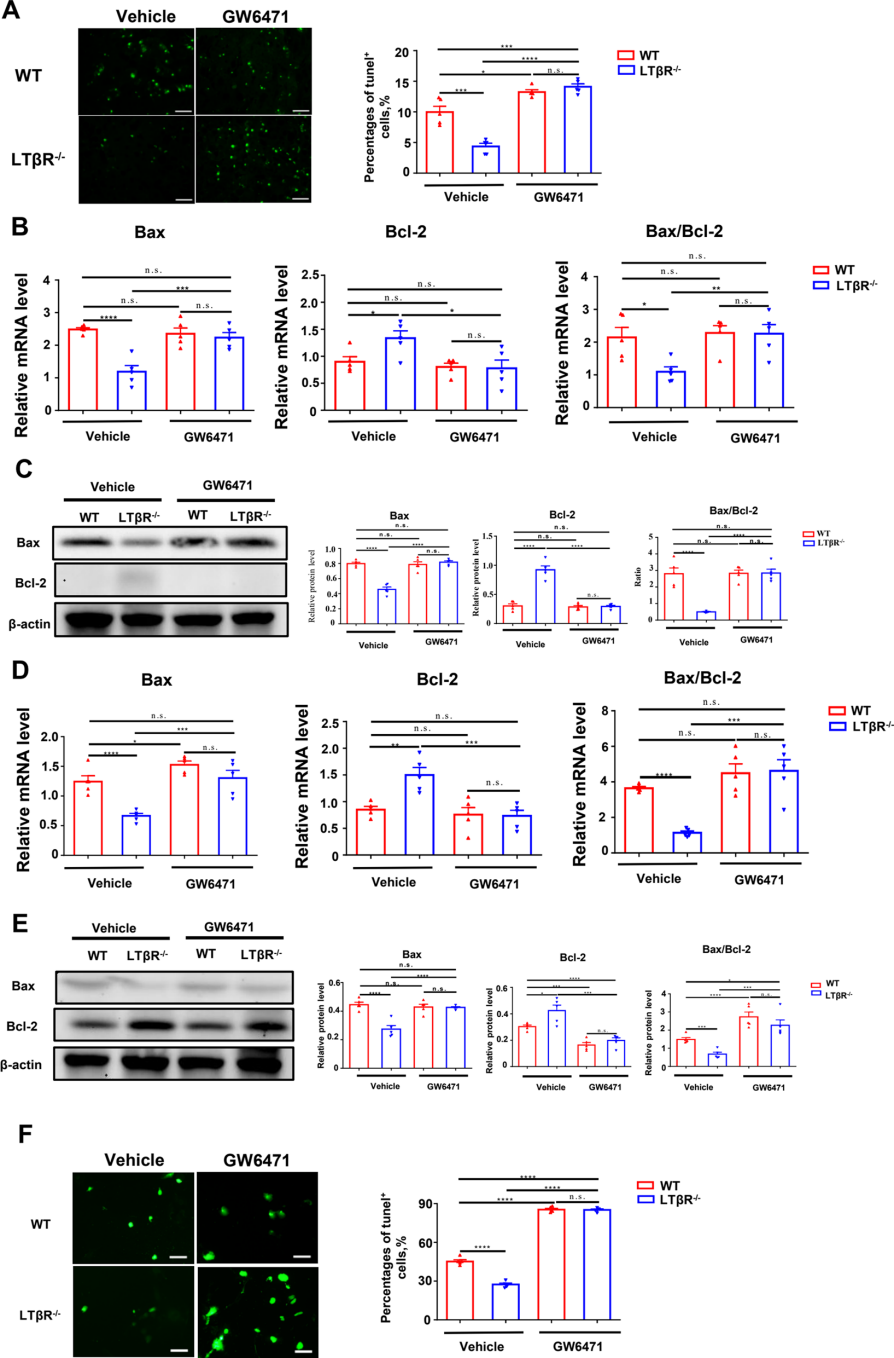
Deletion of LTβR attenuates I/R-induced apoptosis via PPARα. **A** Representative images of TUNEL stained of kidneys from WT mice and LTβR^−/−^ mice in the control I/R group and GW6471 pretreated group. Relative RNA level (**B**) and protein level (**C**) of Bax and Bcl-2 in kidneys from WT and LTβR^−/−^ mice in the control I/R group and GW6471 pretreated group. Relative RNA level (**D**) and protein level (**E**) of Bax and Bcl-2 in RTEC from WT and LTβR^−/−^ mice’ kidneys in control H/R group and GW6471 pretreated group. **F** Representative images of TUNEL stained of RTEC from WT mice and LTβR^−/−^ mice’ kidneys in the control I/R group and GW6471 pretreated group. N = 5/group. Scale bar, 50 μm. Graph data were presented as mean ± SEM. Each point represents an individual subject. P values were determined by t-test. *p < 0.05, **p < 0.01, ***p < 0.005, ****p < 0.001

**Fig. 8:**
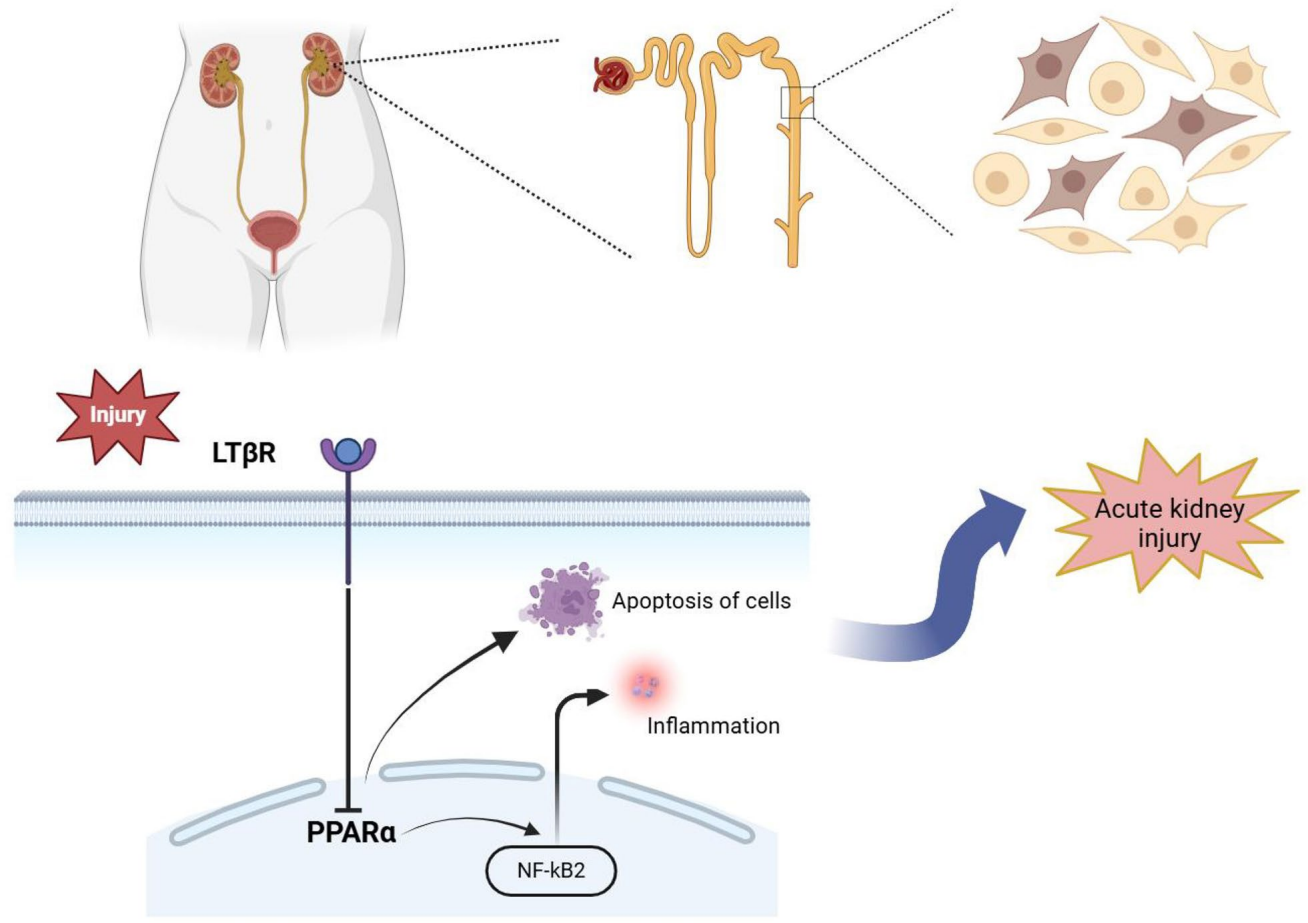
The overall pathway signal pathway of this study

Renal tubular cell apoptosis was one of hallmark of I/R-induced AKI. We previously observed LTβR in protecting against apoptosis. Work from other groups has reported a role of PPARα in anti-apoptotic. Thus, we next to investigate whether PPARα regulated protective effects of LTβR on apoptosis. Our results showed that GW6471 administration significantly increased TUNEL positive cell in LTβR^−/−^ mice following I/R injury, compared with the control group (Fig. 7A). In addition, GW6471 also enhanced the expression of Bax and reduced Bcl-2 expression in LTβR^−/−^ mice (Fig. 7B and C). Consistently, similar trend in Bax/Bcl-2 apoptotic pathway was observed in primary RTEC after H/R injury (Fig. 7D–F). The overall pathway signal pathway of this study shows in Fig. 8.

## Discussion

In this study, we investigated the role and underlying mechanism of LTβR in I/R induced AKI. We found that the depletion of LTβR enhanced kidney function and decreased inflammation in AKI. Results from BMT and primary RTECs suggested that the LTβR expressed in renal tubules may play a dominant role in protecting against AKI. Moreover, our study indicated that depletion of LTβR inhibits downstream non-canonical NF-κB signaling and cell apoptosis by activating PPARα. Future AKI therapies may benefit from the selective therapeutic regulation of LTβR–PPARα interactions.

Although the impact of LTβR on the process of lymphoid organogenesis is widely acknowledged, we further demonstrated that LTβR was an important regulator in preventing AKI. According to recent studies, LTβR has been implicated in a proinflammatory role in various inflammatory illnesses, including rheumatoid arthritis, autoimmune hepatitis, and pancreatitis (Anders et al. 2005; Ishida et al. 2008; Seleznik et al. 2012). Aortic plaque burden and the quantity of lesional macrophages in atherosclerosis are greatly reduced by LTβR deficiency (Grandoch et al. 2015). Nevertheless, the precise role of LTβR in the progression of AKI remains unclear. In this study, we have confirmed that in I/R-induced AKI, renal parenchymal survival and function are improved by the depletion of LTβR. Therefore, during the progression of AKI, blocking LTβR may be a potential treatment strategy. Recent research of Sjögren's syndrome reported that the LTβR-Ig fusion protein partially restored salivary flow and reduced glandular inflammation. LTβR-Ig fusion protein changed lymphocyte trafficking and reduced inflammatory markers in line with its expected mechanisms of action, but not producing considerable clinical efficacy in rheumatoid arthritis or Sjögren's syndrome (St Clair et al. 2018). In the future, it would be interesting to investigate if LTβR-Ig fusion protein can enhance AKI in both clinical and experimental settings.

According to our findings, LTβR in renal tubular epithelial cell rather than inflammatory cells, is a crucial factor in AKI. Prior research has indicated that LTβR is expressed on dendritic cells and macrophages, triggering the production of adhesion molecules and pro-inflammatory mediators (Gubernatorova and Tumanov 2016; Wimmer et al. 2013). An increasing amount of research has revealed that LTβR is expressed on epithelial cells as well and plays a role in the development of inflammation. The generation of epithelial interleukin-23 and defense against epithelial damage in intestinal epithelial cells depend on LTβR signaling (Macho-Fernandez et al. 2015). Furthermore, LTβR activation in gastric epithelial cells led to an expanded pro-inflammatory chemokine milieu, which intensifying the inflammation of the stomach caused by Helicobacter pylori (Mejias-Luque et al. 2017). Consistent with other reports (Seleznik et al. 2016), our findings demonstrated that LTβR was abundantly expressed on renal tubular epithelial cells. To determine whether LTβR in bone marrow-derived cell were involved in AKI, we conducted BMT, and results indicated that the kidney damage and inflammation in LTβR^−/−^ mice receiving bone marrow from WT mice remained mild. Therefore, LTβR in renal tubules have an important effect on the protection of AKI.

Our findings highlight the pathophysiological impact of the LTβR-PPARα axis as a novel pathway in AKI. The nuclear hormone receptor superfamily, PPARα, is mostly expressed in organs involved in metabolism, including the kidney, liver, and heart. In the kidney, PPARα is abundantly expressed in proximal tubules, but little expressed in glomeruli (Abbott et al. 2010). Our study revealed that PPARα expression was stimulated by LTβR deficiency. Furthermore, administration of the PPARα antagonist eliminated the protective effect against AKI in LTβR^−/−^ mice, a comparable alteration was also noted in RTECs following H/R injury. It is reported that activating PPARα prevents post-ischemic contractile dysfunction in hypertrophied neonatal hearts (Lam et al. 2015), as well as improve hepatic ischemia-reperfusion injury (Cheng and Guo 2021). Furthermore, we discovered that the expression of LTβR was unaffected by the PPARα antagonist. Therefore, our research has shown that LTβR is the upstream negative regulator of PPARα.

Three distinct routes of NF-κB activation have been identified: classical, non-classical, and atypical (Viatour et al. 2005). The non-classical NF-κB pathway in immune systems and other cell types is preferentially activated by LTβR activation (Lotzer et al. 2010; Dejardin et al. 2002). The non-classical NF-κB pathway was primarily involved in the development of homeostatic chemokines and proinflammatory mediators, which are involved in the recruitment of inflammatory cells in lymphoid organogenesis and inflammatory diseases (Madge et al. 2008; Dejardin et al. 2002). Furthermore, our findings demonstrated that LTβR deficiency decreased inflammatory cell infiltration in AKI and prevented the development of the non-classical NF-κB pathway. The majority of earlier research focuses on the modifications to the PPARα-regulated classical NF-κB pathway (Glauert et al. 2008). We discovered that after receiving PPARα antagonist therapy, the expression of NF-κB2, Relb, and NIK rose. Therefore, the non-classical NF-κB pathway was also regulated by PPARα in RTECs.

Due to their great susceptibility to apoptosis, renal tubules are involved in both the initial and ongoing damage of ischemia AKI (Havasi and Borkan 2011). It is reported that apoptosis is also regulated by LTβR signaling in embryonic fibroblasts (Lovas et al. 2008), and LTβR–Ig treatment reverses the enrichment of the apoptotic signature in alveolar epithelial cells (Conlon et al. 2020). Moreover, the data available currently indicates that PPARα exerts anti-apoptotic effect, particularly in diseased conditions (Roberts et al. 2002). Our findings showed that apoptosis triggering factor levels were decreased when LTβR was deficient, and this effect was reversed by PPARα antagonist. The LTβR-PPARα axis may inhibit cell apoptosis, thereby providing protection against AKI.

One of the limitations of this study is the inability to identify which of the two LTβR ligands, LTα1β2 or LIGHT, is responsible for activating LTβR and downstream signaling. In addition, we need to utilize tubular cell specific LTβR knockout mice in future to directly confirm that the observed reduction in damage of AKI is attributable to tubular LTβR expression. Nevertheless, our findings demonstrated that the loss of LTβR in RTECs in vitro recapitulates the in vivo observations of PPARα activation and reduced inflammatory responses.

## Conclusions

In summary, our data indicate that deficiency in LTβR improve kidney damage and inflammation in AKI through activation of PPARα and inhibition of downstream non-classical NF-κB pathway. Thus, blockade of LTβR may be an attractive therapeutic option to protect against AKI.

## Supplementary Information

Additional file 1.

## Data Availability

The datasets in this study are available from the corresponding authors upon reasonable request.

## Abbreviations

AKI: Acute kidney injury
Bcl-2: B-cell lymphoma-2
Bax: Bcl-2-Assoiated X protein
BMT: Bone marrow transplantation
BUN: Blood urea nitrogen
H/R: Hypoxia/rreoxygenation
IKKα: Inhibition κB kinase alpha
IL: Interleukin
IRI: Ischemia reperfusion injury
KIM-1: Kidney injury molecule-1
LTβR: Lymphotoxin beta receptor
MCP-1: Monocyte chemoattractant protein-1
NF-κB: Nuclear factor-kappa B
NIK: Nuclear factor-kappa B induced kinase
Ngal: Neutrophil gelatinase-associated lipocalin
PAS: Periodic Acid-Schiff
PPARα: Peroxisome proliferator activated receptor alpha
RNA: Ribonucleic acid
RNA-seq: RNA-sequencing
RTEC: Renal tubule epithelial cells
RT-qPCR: Real time-quantitative polymerase Chain Reaction
Scr: Serum creatinine
TNF-α: Tumor necrosis factor-alpha
TUNEL: TdT-mediated UTP Nick-End labeling
WT: Wild type
