# Correction: Deletion of lymphotoxin-β receptor (LTβR) protects against acute kidney injury by PPARα pathway

**DOI:** 10.1186/s10020-025-01381-5

**Published:** 2025-11-03

**Authors:** Zufeng Wang, Yichun Cheng, Jiahe Fan, Ran Luo, Gang Xu, Shuwang Ge

**Affiliations:** 1 Department of Nephrology, Tongji Hospital, Tongji Medical College, Huazhong University of Science and Technology, No.1095 Jiefang Road, Wuhan, 430030 China; 2 Department of Nephrology, The First Affiliated Hospital of Guangxi Medical University, Nanning, 530021 China

**Correction: Molecular Medicine 30, 254 (2024)**

**https://doi.org/10.1186/s10020-024-01026-z**

In this article (Wang et al. 2024), the affiliation ‘Department of Nephrology, Tongji Hospital, Tongji Medical College, Huazhong University of Science and Technology, Wuhan, China’ for Author Zufeng Wang was missing.

The original article has been corrected.
