# Supplementary material for: Deletion of lymphotoxin-β receptor (LTβR) protects against acute kidney injury by PPARα pathway
Source: Mol Med. 2024 Dec 20;30:254. doi: 10.1186/s10020-024-01026-z (PMC11661049; doi:10.1186/s10020-024-01026-z)
Supplement: Supplementary file 1 — Additional file 1. [file 10020_2024_1026_MOESM1_ESM.pdf]

Table S1. The baseline data of MCD and AKI patients included in this study.

	AKI(n=5)	MCD(n=5)	P
Male(%)	60.0	60.0	1.000
Age	41.0±8.5	38.8±14.2	0.774
Albumin(g)	26.7±5.8	18.9±3.5	0.034
Scr(umol/L)	241.2±62.6	76.8±5.0	<0.001
BUN(umol/L)	17.8±6.6	6.0±1.1	0.004
Proteinuria(g/24h)	3.8±4.1	8.1±5.3	0.199

MCD: minimal change disease; AKI: acute kidney injury; Scr: serum creatinine; BUN: blood urea nitrogen.

Table S2. Primers for PCR

Target	Forward primer (5'–3')	Reverse primer (5'–3')
Mouse GAPDH	GGAAGCTTGTCATCAATGGAAATC	GCGCGATAAATTACGGGCAG
Human β-actin	AAGGTGAAGGTCGGAGTCAA	AATGAAGGGGTCATTGATGG
Mouse LTβR	TGGTGCCCCCTTATCGCATA	TGCATACCGCAAAGACAAACTC
Human LTβR	GGCACCTATGTCTCAGCTAAAT	GTAGTTCCAGTGCTCGTTGTAG
Mouse Ngal	CAGAGCTACAATGTGCAAGTGGC	CAGCTCCTTGGTTCTTCCATACA
Mouse KIM-1	CTGGAATGGCACTGTTGACATCC	GCAGATGCCAACATAGAAGCCC
Mouse PPARα	GAGAATCCACGAAGCCTACCTG	GACCTCTGCCTCTTTGTCTTCG
Mouse NF-κB2	CGGTGGAGACGAAGTTTATTTGC	TCATCTTGTGATAGGGCGGTGT
Mouse NIK	CTCGCTGGGTCAGCTCATAAAG	GAATGTAGTCCCCTGTGAGCAA
Mouse RelB	GAATTGACCCCTACAATGCTGG	GTGCTGAACACCACGGATATGT
Mouse Bax	GCCTTTTTGCTACAGGGTTTCAT	TATTGCTGTCCAGTTCATCTCCA
Mouse Bcl-2	GCTACCGTCGTGACTTCGCA	CATCCCAGCCTCCGTTATCC

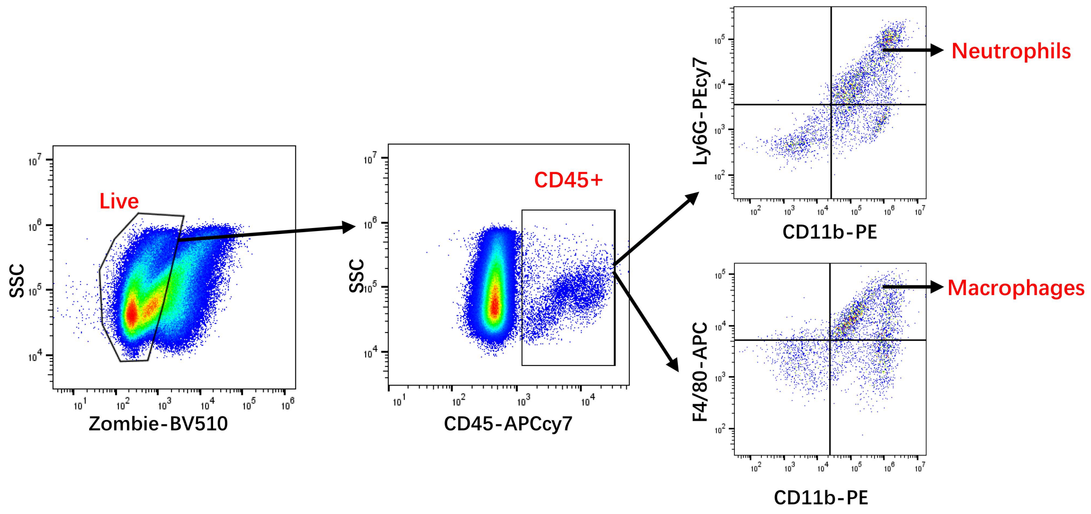

Figure S1.The flowcytometry gating strategy of inflammatory cell.

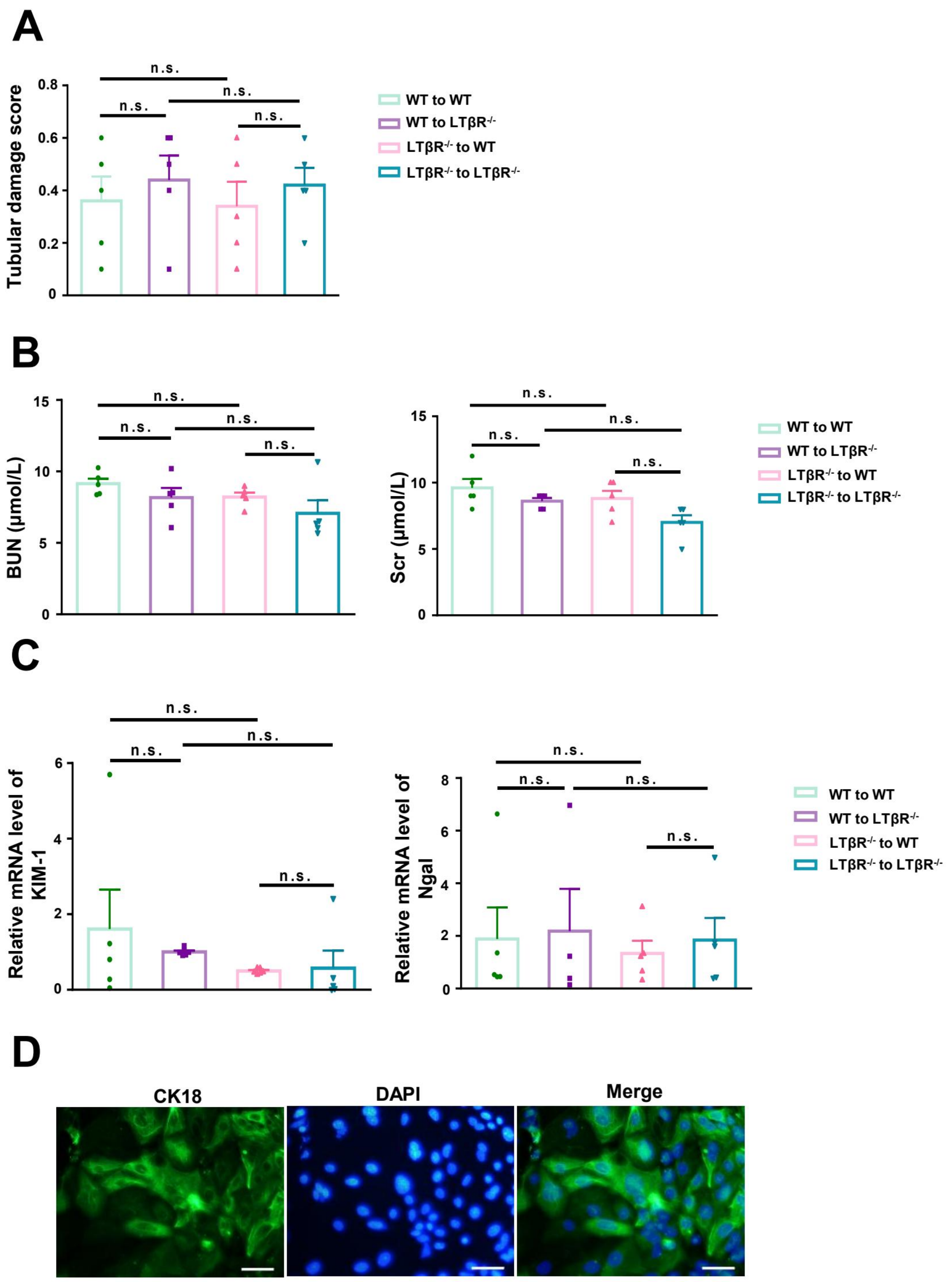

Figure S2. A-C: There is no damage to the kidneys after BMT. A: Tubular damage score of four sham BMT groups. B: Scr and BUN of mice from four sham BMT groups. C. Relative RNA level of NGAL and KIM-1 in kidneys from sham BMT groups. N=5/group. D: Representative images of immunofluorescence staining of CK18 in RTEC. Scale bar, 50 μm. Graph data were presented as mean ± SEM. Each point represents an individual subject. P values were determined by t-test. *p<0.05, **p<0.01, ***p<0.005, ****p<0.001.
